# Patterns and Predictors of Service Use Among Women Who Have Separated from an Abusive Partner

**DOI:** 10.1007/s10896-015-9688-8

**Published:** 2015-03-18

**Authors:** Marilyn Ford-Gilboe, Colleen Varcoe, Marianne Noh, Judith Wuest, Joanne Hammerton, Eman Alhalal, Camille Burnett

**Affiliations:** 1 H37 HSA, Arthur Labatt Family School of Nursing, Western University, 1151 Richmond St., London, Ontario N6A 3C1 Canada; 2 School of Nursing, University of British Columbia, Vancouver, BC Canada; 3 Faculty of Nursing, University of New Brunswick, Fredericton, NB Canada; 4 School of Nursing, University of Virginia, Charlottesville, VA USA

**Keywords:** Intimate partner violence, Unmet need, Access barriers, Help-seeking, Mental health, Chronic pain, Social location

## Abstract

Using baseline data from a survey of 309 Canadian women recently separated from an abusive partner, we investigated patterns of access to health, social, legal, and violence-specific services and whether abuse history and social and health variables predict service use. We compared rates of service use to population rates, and used logistic regression to identify determinants of use. Service use rates were substantially higher than population estimates in every category, particularly in general and mental health sectors. Although women were confident in their ability to access services, they reported substantial unmet need, difficulty accessing services, and multiple barriers. The strongest unique predictors of use varied across service type. Health variables (high disability chronic pain, symptoms of depression and PTSD), low income, and mothering were the most consistent predictors. Service providers and policy makers must account for social location, abuse history, and health status of Intimate Violence (IPV) survivors. Strategies to enhance access to primary health care services, and to create a system of more integrated, accessible services, are required.

Intimate partner violence (IPV), a pattern of physical, sexual, and/or emotional violence by a current or former partner in the context of coercive control, is a significant global problem affecting between 10 and 71 % of women in their lifetimes (Garcia-Moreno et al. 2006), including one in four Canadian women (Clark and Du Mont 2003). IPV has been linked to a wide range of physical and mental health consequences (Carbone-López et al. 2006; Golding 1999; Hill et al. 2009; Mechanic et al. 2008) and accounts for a significant proportion of the global burden of disease for women (Lim et al. 2012). Although the primary social solution to IPV has been for women to leave their abusive partners, little attention has been given to what happens in women’s lives after separation. There is some evidence that life after separation is often complicated; women’s risk of continued violence increases during this time (Alsaker et al. 2007; Brownridge 2006; Mechanic et al. 2000) and they experience changes in their personal, social, and economic resources that may affect their health and quality of life (Ford-Gilboe et al. 2009; Wuest et al. 2003). Women’s social networks may not provide needed support, but, instead, may undermine women’s healthy decision-making by minimizing the abuse, blaming the women, and/or maintaining secrecy regarding IPV (Barnett 2001; Lempert 1997; McLeod et al. 2010; Merritt-Gray and Wuest 1995). In the context of high social conflict post-separation, the positive benefits of social support on women’s health are diminished (Guruge et al. 2012).

Although informal support from family members and friends is an important and frequently sought after resource for women who have experienced IPV (Statistics Canada, 2011a), there is evidence that women also seek help from services in varied sectors (DuMont et al. 2005) and that these services protect them from abuse (Liang et al. 2005; Sabina and Tindale 2008). In a recent analysis of data from the Canadian General Social Survey (GSS) (Barrett and Pierre 2011), women who had experienced IPV in the past 5 years reported using an average of two services to help them deal with the violence. However, some services were used more than others, as 6 % sought assistance from police or court-based services and 39 % sought counseling. Using a greater number of services was associated with particular characteristics of women (e.g., higher income, Aboriginal, disabled) and with increased severity of violence, the latter finding also being consistent with Ansara and Hindin’s (2010) analysis of the GSS. These findings suggest that use of services *in general* by IPV survivors is a complex process that may be shaped by many factors. Understanding the context of service use in varied sectors is important to developing effective services and policies for women who have experienced IPV and, yet, the determinants of using *particular* types of services have not been well studied.

## Use of Services in Different Sectors

Growing evidence of the substantial and often disabling health problems experienced by women with histories of IPV led to studies focused on health service use in this population. In U.S. based studies, women have been found to use a variety of health services to cope with the impact of IPV (Goodman et al. 2003; Hathaway et al. 2000; Shannon et al. 2006) and to make more visits to health care providers than women without an IPV history (Bonomi et al. 2009; Duterte et al. 2008; Plichta 2007; Rivara et al. 2007; Snow-Jones et al. 2006). Furthermore, women who have experienced recent or current abuse have been found to use more services (Bonomi et al. 2009; Rivara et al. 2007; Snow-Jones et al. 2006). Consistent with findings related to service use in general, greater severity of abuse has been associated with higher health service use in studies conducted in Canada (Ansara and Hindin 2010), the U.S. (Coker et al. 2004; Duterte et al. 2008), and Australia (Hegarty et al. 2013).

Disabling mental and physical health problems place women at greater risk of IPV (Brownridge 2006) and poor mental health has been found to increase women’s risk of returning to an abusive relationship after they have separated (Alhalal et al. 2012). Among female IPV survivors, living with a disability has been associated with greater use of services in general (Barrett and Pierre 2011). However, research has not explored how the mental and physical health of women who have experienced IPV shapes their use of services, particularly those outside of the health care system.

Use of social and violence specific services has received considerable attention in research on help-seeking among women who have experienced IPV. For example, it has been estimated that one in four women accessing welfare services have been exposed to IPV within the past year (Kimerling and Baumrind 2004; Lown et al. 2006). While only 11 % of Canadian women who had experienced IPV in the past 5 years reported accessing a domestic violence (DV) shelter and 17 % used a DV crisis line (Barrett and Pierre 2011), less is known about women’s use of a wider range of domestic violence services, particularly after separation.

Use of social and violence-specific services among women who have experienced IPV has been associated with some aspects of women’s social locations. For example, female survivors who were *recent* immigrants were less likely to access social services, including violence-specific services, than non-immigrant women or immigrant women who had lived in Canada for 10 years or more (Hyman et al. 2006). Greater use of these services has also been associated with living in an urban setting in some studies (Shannon et al. 2006) and with rurality in others (Barrett and Pierre 2011). However, how women’s social locations affect help-seeking across sectors is still poorly understood.

### Service Access Barriers and Unmet Need

Despite high service use, women exposed to IPV are more likely than those without exposure to have unmet care needs and face barriers in accessing services (Plichta 2007), particularly mental health services (El-Khoury et al. 2004; Lipsky and Caetano 2007). General service barriers, identified in previous research, include: negative or insensitive attitudes of service providers (Kramer et al. 2004; Paranjape et al. 2006; Robinson and Spilsbury 2008), limited finances (Paranjape et al. 2006; Wilson et al. 2007), inappropriate referrals (Robinson and Spilsbury 2008), transportation problems (Annan 2008), lack of privacy (Fugate et al. 2005; Robinson and Spilsbury 2008), and fear that the abuser may find out and retaliate (Plichta 2007; Robinson and Spilsbury 2008). Whether and how these barriers continue to affect women’s service use after separation remains unknown. Furthermore, the extent to which these barriers vary by service type remains unclear.

### Key Gaps in Understanding Service Use and IPV

Collectively, studies of service use among women who have experienced IPV tend to focus on either *general services* or *one specific type of service*, and have not addressed service use or level of unmet need across varied sectors. With the exception of studies of health services, largely conducted in the U.S., few distinctions have been made between women who are living with, versus separated from, an abusive partner. Thus, in the transition out of an abusive relationship, the broad range of services women access, including those which extend beyond health services, along with access barriers and level of unmet need, have not been systematically studied, nor have factors that shape service use been identified. This is particularly true in Canada where core health and social services are publicly funded and there is a mandate to promote universal access for all people. Given differences in Canadian and U.S. health and social service systems, patterns of service use, levels of unmet need, and the barriers faced by women may differ across these different system contexts.

In this paper, we address these gaps by presenting a comprehensive analysis of patterns of access to a broad range of services in health, social, violence-specific, and legal sectors among Canadian women who had separated from an abusive partner in order to better understand the context of service use. First, we describe rates of use and unmet need for 26 different services. Where possible, we compare rates of use in our sample to those of Canadian women in general, allowing for a more nuanced understanding of variations in the magnitude of specific service use among women who are transitioning out of an abusive relationship. Next, we describe access barriers and levels of unmet need in an effort to make visible the sometimes hidden gaps and weaknesses which exist in more socialized service delivery systems. Finally, we examine how women’s abuse histories, health, and social locations predict use of different types of services in an effort to better understand what “drives” service use in different sectors. A broad, contextual understanding of women’s service use is critical to developing services relevant to women’s health, social, economic, and legal needs after they separate from abusive partners. Beyond this, our analysis may offer insights regarding how publicly funded, socialized systems present in many countries, shape women’s access to, and use of, needed services.

## Method

This analysis uses baseline data from the Women’s Health Effects Study (Ford-Gilboe et al. 2009), a prospective, longitudinal survey of 309 adult, English-speaking women who had been separated from an abusive partner for an average of 20 months when they entered the study. The diverse community sample of women was recruited from three Canadian provinces—Ontario (ON), New Brunswick (NB), and British Columbia (BC)—through newspaper advertisements, media coverage, posters in community settings, and referrals from social service agencies (Ford-Gilboe et al. 2009). Exposure to IPV in the relationship with the woman’s ex-partner was confirmed using a modified version of the Abuse Assessment Screen (AAS) (Parker and McFarlane 1991), which included four items (one each for physical abuse, forced sex, fear of partner, and experiences of coercive control). An affirmative response to at least one of four screening questions was considered positive for IPV.

Through face-to-face structured interviews and health assessments, information about demographic characteristics, service utilization, and personal, social, and economic resources was collected by trained interviewers using standardized measures and survey questions. Registered Nurses conducted health assessments, which included in-depth abuse and health histories. A detailed safety protocol, designed to support women’s physical and emotional safety, was used to guide all interactions with the research team. This protocol included structured guidelines for: a) conducting a safety risk assessment at intake and updating it at each contact; b) obtaining women’s preferences about communication with the team, including a plan for dealing with potentially unsafe situations before, during, or after an interview; c) debriefing all women about the potential for a stress response after each interview, with examples of common responses and management strategies; and, d) suicide assessment and follow up for women who reported suicidal ideation. Since interviews took place in community settings, including women’s homes, guidelines were also used to promote the personal safety of interviewers before, during, and after conducting interviews. For example, interviewers were directed to negotiate the interview location based on the woman’s current safety risk; check-in with a contact person before and after each interview; be observant of the environment; carry a charged cell phone at all times; and to end an interview if they felt unsafe. Regular sessions were held with interviewers to debrief their experiences and to reinforce the safety protocol.

### Measurement

#### Service Use and Unmet Need

Past month service use was measured using women’s self-reports of: 1) whether they had used specific services (*yes*/*no*); 2) number of visits made to each; and, 3) if they needed, but were unable to access each service (*yes*/*no*). Data were collected about five major categories of service use: 1) seven *general health* services, including family doctors, dentists, and hospitalizations; 2) five *mental health* services, including psychiatric, psychological, and support group services; 3) six *social services*, including food banks and social assistance; 4) five *violence-specific services*, including abuse help lines, shelters, and counseling for violence; and, 5) three *legal services*, including police and legal aid. Use of social assistance, disability benefits, and employment insurance was measured using women’s reports of receiving such benefits in the previous 6 months. Women were also asked whether they were living in private market or social housing. Women’s use of each service in the past month was coded as 0 (*no service use*) or 1 (*used at least once*). For each of the five categories of service, total service use scores were created by summing the values (0,1) assigned to each service in that category and used to group women into non-users (score of 0) or users (score of 1 or more) of services.

#### Capacity to Access Services and Barriers

Women were asked to assess their general capacity to access services: 1) past year contact with someone who knows about community services and how to access them (0 = *no*, 1 = *yes*); 2) level of confidence in ability to access services from *not at all confident* (1) to *very confident* (4); and, 3) past year difficulty getting the support needed from the system from *not at all difficult* (1) to *very difficult* (4). If respondents answered *yes* to 1 above or reported difficulty getting support, they were asked who they knew and which difficulties, respectively, as follow up questions.

#### Social Location

Women’s ages in years, years of education (<*12 years* = 0, *12 or more years* = 1), and the presence of at least one dependent child (<18 years of age) at home, were measured using women’s self-reports. Women’s residence was classified as rural if they lived in an area with a population of less than 10,000 that was either not adjacent (i.e., more than 30 min driving time) to an urban center of 50,000 or more or, if adjacent, where accessible public transit to the urban center was not available 7 days a week. This approach is consistent with Statistics Canada’s Rural and Small Town (RST) designation (Bollman and Clemenson 2008) but challenges the assumption that proximity to an urban center is equivalent to accessibility, a point that is particularly salient for people living on lower incomes who may not have access to their own means of transportation. Women’s reports of annual household income were classified as *low income* (1) or *not low income* (0) using Statistics Canada’s low-income cut-offs (LICOs), a widely used proxy for poverty which is based on the proportion of income spent on basics needs, such as clothing, food, and shelter, and adjusted for family size and local cost of living (Statistics Canada 2012).

#### Abuse History

IPV Severity, the intensity of *past* abuse directed toward a woman by the intimate partner she recently left (i.e., the index partner), was measured using the 30-item Index of Spouse Abuse (ISA) (Hudson and McIntosh 1981). On the ISA, women rated the frequency of 19 physical and 11 non-physical abusive acts directed toward them by the abusive partner from whom they had separated ranging from *never* (0) to *very frequently* (4). Standard scoring was applied to derive separate scores for physical and non-physical abuse by weighting individual items for severity and summing these weighted scores, for a possible range of 0 to 100 (Hudson and McIntosh 1981). These continuous scores were used in all analyses. The presence of ongoing (i.e., current) abuse was measured by asking women a single question (*Is the abuse ongoing*?) after they completed the ISA, with responses coded as *no* (0) or *yes* (1). Women were also asked to indicate the number of intimate partners who had been abusive toward them in their lifetimes, as well as whether they had experienced abuse as children (*no* = 0, *yes* = 1).

#### Health Status

Four measures of women’s health were used in this analysis. Women’s physical health was measured using the Physical Component Summary Score (PCS) of the Short-Form 12 Health Survey Version 2 (SF12v2) and the Chronic Pain Grade (Von Korff et al. 1992). The SF12v2, a brief self-report measure of functional health status, which is an alternative to the SF-36 (Ware et al. 1996), has been used extensively to measure the health of various groups of adults. Participants rate various aspects of their health in the past 4 weeks on a 3-point (1 = *yes limited a lot*, 2 = *yes limited a little*, 3 = *no*, *not limited at all*) or 5-point (1 = *not at all* to 5 = *extremely*) Likert scale, depending on the item. The Physical Component Summary score (range 0–100) is computed using a standardized scoring procedure in which weighted scores on subscales are summed, with a score of 50 equivalent to the population mean. In this study, scores of 50 or greater were categorized as *higher physical functioning* (1). Scores of less than 50 were coded as the reference category (0 = *lower physical functioning*).

The 7-item Chronic Pain Grade (CPG) scale (Von Korff et al. 1992) was used to measure disability related to chronic pain (i.e., pain grade). Participants were asked to rate the interference of their pain, the worst pain intensity, and number of days lost from usual activities due to pain and assigned disability points based on their scores. Four categories of disability-related chronic pain were created using a procedure which combines the total number of disability points and pain intensity scores: Grade 0 (*pain free*); Grade I (*low disability*, *low intensity*); Grade II (*low disability*, *high intensity*); Grade III (*high disability*, *moderately limiting*); Grade IV (*high disability*, *severely limiting*). Consistent with guidelines for the scale, pain grades of 0, 1, and 2 were classified as *No or Low disability chronic pain* (0), while grades 3 and 4 were classified as *High disability chronic pain* (1).

Mental Health was measured using established self-report measures of symptoms of depression and PTSD, two common and often debilitating mental health problems experienced by IPV survivors (Golding 1999; Hedtke et al. 2008). First, the 20-item Center for Epidemiologic Studies-Depression (CES-D) Scale (Radloff 1977) was used to measure depressive symptoms. Women’s ratings of symptom frequency in the past week on a 4-point scale from *rarely or none of the time* (1) to *most of the time* (4) were summed to produce total scores (range 0–60). The standard cut score was used to classify scores into two groups: *no or low depressive symptoms* (<16), *moderate to high depressive symptoms* (scores ≥ 16) (Radloff 1977). The Cronbach’s alpha for the CESD in this study was 0.93.

Second, the presence of PTSD *symptomology* was measured using the 17-item Davidson Trauma Scale (DTS) (Davidson 1996; Davidson et al. 1997). Women who identified themselves as having experienced a traumatic event were asked to rate the past week frequency and severity of symptoms (i.e., intrusion, hyper-arousal, avoidance) consistent with DSM-IV diagnostic criteria for PTSD. Separate frequency and severity scores were computed by summing responses to applicable items (range 0–56), while the overall score was created by summing the frequency and severity scores (range 0–136). Cronbach’s alpha was 0.95. A cut score of 40, in combination with minimum score for each symptom cluster, have been shown to reliably predict symptoms consistent with PTSD (Davidson et al. 1997). This scoring was used to create a dichotomous variable: *No or Low PTSD symptoms* (0), *High PTSD symptoms* (1).

### Data Analysis

Descriptive statistics appropriate to the data were used to summarize the study variables. In order to compare rates of service use in our sample to those of the population, we used national population data (or Ontario data if not available) to identify population estimates based on similarity of question, and timing of data collection (i.e., as close to 2004–2005 as possible). When possible, we drew estimates for women of similar age (18–65) to our sample (see Varcoe et al. 2011 for details). Ninety-five percent confidence intervals were used to determine significant differences in rates of use. Multivariate Logistic Regression was used to identify significant determinants of service use, with separate analyses conducted for each type of service. In each analysis, 12 variables, representing three categories of predictors (i.e., social location, abuse history, health) were entered simultaneously into the regression models to estimate the extent to which each independent variable predicted service use, controlling for the effects of all other independent variables. To enhance ease of interpreting the results, categorical variables were used for all predictors with the exception of age and severity of past physical and non-physical abuse. Listwise deletion of 45 cases with values missing on any variable was used, resulting in a final sample size of 264 for the logistic regression analysis. This conservative approach for handling missing data resulted in an adequate sample for conducting this analysis based on an a priori power analysis calculation conducted in *GPower (Faul et al. 2007), where a power of 0.80, alpha of <0.05, and moderate effect size had been stipulated. Given that few studies have examined factors that are associated with service use across multiple domains, we took an exploratory approach to determine best-fit models for each service type. In all analyses, *p* values of <.05 were considered significant.

## Results

### Sample Characteristics

A profile of women’s social locations, abuse histories, and health is provided in Table 1. In order to understand women’s social locations vis-à-vis Canadian norms, selected population estimates for Canadian women of similar age (18–65) are provided. Women were an average of 39 years of age (*SD* = 9.8, range 19–63) and had completed 13 years of education (*SD* = 2.6, range 6–22). Fifty-seven percent were parenting at least one dependent child. Nearly half (45 %) were employed but the majority (59 %) had incomes which placed them below the poverty line. Mean annual household income was $20,391 (*SD* = 17,143, range 0 to 95,000). Our sample is similar to women in the Canadian population with respect to educational attainment, the percentage who lived in a rural area (23 %), and percentage of racialized women (i.e., those reporting visible minority status), but differs on economic challenges (i.e., higher rates of unemployment, social assistance, disability benefits, and low income in our sample). Inclusion of Aboriginal women (7.4 %) was almost double that found in the Canadian population (4 %).

**Table 1:** Demographic characteristics of the sample (*N* = 309) in comparison to population estimates for Canadian women

Variable	WHES sample	Canadian women^a^
Education		
Less than high school High school Some post-secondary	16.6 % 22.2 % 61.3 %	14.5 % 25.0 % 60.5 %^b^
Employment		
Employed Social assistance Disability benefit	45.1 % 31.4 % 10.4 %	57.5 % 4.3 %^c^ 2.7 %
Low income	59.0 %	11.4 %^d^
Rural residence	23.0 %	26.5 %
Visible minority	16.8 %	16.2 %
Aboriginal	7.4 %	4.0 %

^a^Authors’ calculations of raw data (Statistics Canada 1993 and 2006 Census) for women age 18–65 unless otherwise indicated

^b^Statistics Canada (2006a)

^c^General Social Survey (Statistics Canada 2003)

^d^Collin and Jensen 2009)

Women reported substantial histories of IPV and lifetime violence. For example, 66.3 % had experienced abuse as children and 59 % had more than one abusive partner in their lifetimes. Although separated for a mean of 20 months, half (49 %) reported that the abuse was ongoing. Only 16 % of women reported that they had stayed in a shelter in the first 6 months after separation.

Health issues were significant among women in this sample, with 82 % reporting at least one active medical diagnosis. Furthermore, 48.5 % of women were living with high disability chronic pain, 73 % had moderate to high levels of depressive symptoms, and 48 % had symptoms consistent with PTSD. Fifty-six percent had levels of physical functioning which were worse than population norms for women of similar age.

### Rates of Service Use and Unmet Need

Past month rates of service use and inability to access services (i.e., unmet need) are presented in Table 2. Women had considerable contact with services across a variety of sectors, with 94 % using at least one service in the past month. On average, women used four different services (out of a possible 26) and made a total of 11 visits to those services in the past month. For all services for which we could make comparisons to the population, service use was significantly higher in our sample (range 1.25 to 292 times higher, 95 % CI, *p* < 0.001). For services in which unmet need is reported, the highest rates of inability to access needed services were observed for a dentist (21 %), psychologist (11.3 %), family doctor (11 %), medical specialist (8.7 %), and psychiatrist (8.4 %).

**Table 2:** Past month service use and unmet need reported by category (*N* = 309)

Specific service	WHES sample					Comparison service use		Difference in service use
	% who used service (*n*)	No. of visits			% unable to access (*n*)	Mean visits for users/	% who used service	Rate of increased use in sample
		Mean	SD	Range				
General health								
Family doctor/general practitioner	56.3 (174)	1.06	1.33	0–10	11.0 (34)	0.35^a^		3.8
Walk-in clinic	22.0 (68)	0.42	1.04	0–10			2.6 %^a^	8.5
Dentist	21.4 (66)	0.31	0.72	0–5	21.0 (65)	0.12^a^		2.6
Emergency department	13.9 (43)	0.24	0.88	0–10		0.012^a^		20.0
Public health nurse	6.1 (19)	0.20	1.79	0–30	1.0 (3)	0.036^a^		5.5
Hospitalizations	5.2 (16)	0.32	2.20	0–30		0.03 nights^b^		10.7
Medical specialist	15.5 (48)	0.35	1.07	0–8	8.7 (27)			
Total	77.3 (239)							
Mental health								
Support group	25.2 (78)	1.28	3.80	0–30	6.1 (19)			
Psychiatrist	13.6 (42)	0.34	1.86	0–30	8.4 (26)		0.19 %^a^	71.6
Social worker	11.7 (36)	0.24	0.81	0–6	1.3 (4)		0.04 %^a^	292
Psychologist	10.0 (31)	0.23	0.96	0–12	11.3 (35)	0.02^b^		11.5
Addictions counselor	6.5 (20)	0.19	1.03	0–14	1.0 (3)		0.5 %^c^	13
Total	42.7 (132)							
Social services								
Food bank	22.3 (69)	0.37	0.86	0–4	4.9 (15)		1.66 %^d^	13.4
Social assistance worker	33.7 (104)	0.29	0.43	0–1			4.3 %^e^	7.8
Disability benefits worker	11.3 (35)	0.10	0.30	0–1			6.6 %^f^	1.7
Social housing	15.2 (47)	0.15	0.36	0–1				
Child protection worker	6.8 (21)	0.15	0.81	0–8	1.0 (3)		0.123 %^g^	56.3
Employment insurance	9.1 (28)	0.08	0.32	0–2			7.28 %^h^	1.25
Total	59.9 (185)							
Violence specific services								
Woman abuse helpline	11.0 (34)	0.23	0.83	0–6				
Sexual assault/rape crisis centre	3.2 (10)	0.15	1.50	0–25				
Domestic violence (DV) advocacy/counseling	33.0 (102)	1.26	2.84	0–20				
DV shelter	1.6 (5)	0.26	2.50	0–30				
Transition home/2nd stage housing	9.4 (28)	2.46		0–30				
Total	40.8 (126)							
Legal services								
Legal aid	10.7 (33)	0.16	0.55	0–4	4.9 (15)	0.02^i^		8.0
Police services	13.9 (43)	0.25	0.76	0–5	0.3 (1)		0.46 %^j^	30.2
Victim services	10.7 (33)	0.21	0.80	0–8	2.3 (7)			
Total	27.5 (85)							

^a^Canadian Community Health Survey (Statistics Canada 2005a)

^b^Canadian Institute for Health Information (CIHI) 2005

^c^Canadian Addiction Survey (Canadian Executive Council on Addictions 2004)

^d^Hunger Count Survey (Canadian Association of Food Banks 2005)

^e^General Social Survey (Statistics Canada 2003)

^f^Participation and Activity Limitation Survey (Statistics Canada 2006b)

^g^Trocomé et al. (2005)

^h^CANSIM (Statistics Canada 2007)

^i^Legal Aid Survey (Statistics Canada 2005b)

^j^General Social Survey Cycle 23 - Victimization (Statistics Canada 2009)

Remarkably, 77 % of participants visited a general health provider at least once in the past month. Over half (56.3 %) saw a family doctor and made an average of 1.06 visits, a rate 3.8 times higher than in the population; another 11 % reported that they needed to see, but could not access, a family doctor. Slightly more than one in five women used a walk-in clinic (22 %) and saw a dentist (21 %) in the past month; another 21 % needed to see but could not access a dentist. Almost 14 % of participants accessed care in the Emergency Room in the past month, a rate 20 times higher than in the general population.

Mental health service use was also high. Of the 43 % of women who saw a mental health provider, a peer support group was most often used, but rates of accessing professional mental health services were also high, including visits to psychiatrists (13 %), social workers (12 %), psychologists (10 %), and addiction counselors (6.5 %). The greatest unmet need in this category was related to accessing psychiatrists (8 %) and psychologists (11 %).

Sixty percent of women in the study used a social service within the past month, including a staggering 22 % of women who visited a food bank (a rate 13 times greater than the general population) and another 5 % who needed a food bank but could not access one. Thirty-four percent of women accessed social assistance, a rate nearly eight times that of the Canadian population.

Although women had been separated from their partners an average of 20 months, one in three sought help from a domestic violence advocacy or counseling service in the last month and another 11 % used a helpline. The rate of unmet need is not known as this question was not asked in relation to violence-specific services. With respect to legal services, 14 % of women called the police and over 10 % of women accessed government funded legal services at least once in the past month. Another 6 % of women said they needed legal aid but could not access such services.

### Women’s Capacity to Access Services and Barriers

The vast majority of women (80 %) were confident in knowing where to go for services, with 92 % reporting that they knew someone who could help them navigate the system, most often a friend (50.8 %), family doctor (46.9 %), or a social worker (40.8 %). Despite this, 65 % reported difficulty accessing the services they needed. The percentage of women who reported each of seven service access barriers ranged from a low of 16.2 % for lack of response from the provider to 50 % for inability to pay for a service (Table 3). Women living on low incomes were more likely than those who were not living on low income to report barriers in five of the seven areas measured.

**Table 3:** Frequency of service access barriers: total and by income (*N* = 309)

Service access barrier	Frequency of access barriers (%)			Chi square
	Sample	Not low income	Low income	
Can’t afford service	50.0	38.2	59.6	13.3*
Placed on waiting list	42.4	28.5	52.2	16.8*
No transportation	32.4	15.4	43.8	26.8*
No information available about service	26.2	28.5	25.3	0.38
No child care	18.8	8.9	25.3	12.8*
No service in area	17.8	11.4	21.9	5.6*
Lack of response from service provider	16.2	12.2	18.5	2.2

*p < .05

### Predictors of Service Use

In order to take into account the concurrent effects of social location, abuse history, and health on the odds of using each type of service, all predictors were simultaneously entered into separate logistic regression analyses for each service type (Table 4). All models were significant, although the strongest unique predictors of service use varied by service type. Only high disability chronic pain predicted general health service use (*OR* = 2.84). In contrast, three variables (age, moderate to high depressive symptoms, and high disability chronic pain) predicted the use of mental health services, with the strongest odds observed for high disability chronic pain and depressive symptoms (*OR* = 2.55, 2.08, respectively). Low income and having dependent children were each associated with increased odds of using social services (*OR* = 6.31, 2.52, respectively), while having less than 12 years of education was associated with reduced odds of using social services (*OR* = 0.40). With respect to the use of violence-specific services, low income, ongoing abuse, and moderate to high depressive symptoms were each associated with increased likelihood of use (*OR* = 1.90, 1.90, 1.66, respectively). Finally, legal service use was associated with having dependent children at home (*OR* = 2.04) and with high PTSD symptomology (*OR* = 2.08).

**Table 4:** Logistic regression of five types of service use on social location, abuse history, and health variables (*n* = 264)

Predictors	General health			Mental health			Social			Violence specific			Legal		
	*B*	*SE*	*OR*	*B*	*SE*	*OR*	*B*	*SE*	*OR*	*B*	*SE*	*OR*	*B*	*SE*	*OR*
Social location															
Age	0.01	0.02	1.01	0.03^*^	0.02	1.03	0.01	0.02	1.01	−0.02	0.02	0.98	0.00	0.02	1.00
Rural location	0.57	0.38	1.77	−0.47	0.31	0.63	−0.22	0.35	0.81	0.09	0.32	1.01	0.21	0.33	1.23
Low Income	0.23	0.33	1.26	0.11	0.30	1.12	1.84^***^	0.31	6.31	0.64^*^	0.30	1.90	−0.02	0.32	0.98
Education (12 < years)	0.19	0.33	1.22	0.15	0.30	1.17	−0.92^**^	0.34	0.40	0.37	0.30	1.45	0.19	0.32	1.21
Dependent children	−0.54	0.33	0.59	0.48	0.29	1.61	0.92^**^	0.32	2.52	0.38	0.28	1.46	0.71^*^	0.32	2.04
Abuse history															
Past IPV severity (physical)	0.01	0.01	1.01	−0.00	0.01	1.00	0.00	0.01	1.00	0.00	0.01	1.00	0.01	0.01	1.01
Past IPV severity (non-physical)	0.01	0.01	1.01	0.00	0.01	1.00	−0.01	0.01	0.99	0.01	0.01	1.01	0.00	0.01	1.00
Ongoing abuse from ex-partner	−0.27	0.32	0.76	0.50	0.28	1.65	−0.06	0.32	0.94	0.64^*^	0.28	1.90	0.31	0.30	1.36
Health status															
physical functioning	0.16	0.34	1.17	0.26	0.31	1.30	−0.10	0.34	0.91	−0.13	0.31	0.87	−0.07	0.33	0.94
Depressive symptoms	0.22	0.36	1.25	0.73^*^	0.34	2.08	0.22	0.37	1.24	0.51	0.34	1.66	0.07	0.38	1.08
PTSD symptoms	−0.04	0.34	0.96	0.46	0.30	1.59	0.30	0.34	1.34	0.37	0.29	1.45	0.73^*^	0.33	2.08
High chronic pain disability	1.04^**^	0.40	2.84	0.94^**^	0.32	2.55	1.01^**^	0.37	2.74	0.28	0.31	1.32	0.24	0.33	1.27
Constant	−0.33	1.08	0.72	−3.24^***^	0.98	0.04	−0.80	1.06	0.45	−2.19^*^	0.94	0.11	−2.81	1.04	0.06
Chi-square	21.969*			40.457***			91.474***			38.109***			21.038*		
Nagelkerke *R* ^2^ statistics	0.12			0.18			0.38			0.17			0.11		

*p ≤ .05, **p ≤ .01, ***p ≤ .0

## Discussion

Separating from an abusive partner is a significant transition involving many challenges, including the emotional and physical health impacts of abuse, the financial and emotional upheaval that occurs with separation, the responsibility of single parenting, trying to find affordable housing, and paying for legal costs associated with custody/access issues. The reality of abuse and harassment persists for many women, and sometimes escalates post-separation, compounding women’s stress (Wuest et al. 2003). Our results show that women were confident in knowing how to access services, had support available to help them, and used services at rates that were consistently and markedly higher than women in the general population. These findings are consistent with the position that women who have separated from abusive partners are not passive victims but actively seek varied assistance to improve the conditions of their lives (Duterte et al. 2008; Goodman et al. 2003; Robinson and Spilsbury 2008). Although the use of health and social services is known to be higher among women who have experienced IPV compared to those who have not (Bonomi et al. 2009; Domino et al. 2005), our findings extend current knowledge beyond service use by Canadian women who have experienced IPV (Ansara and Hindin 2010; Barrett and Pierre 2011) to those who have *separated* from an abusive partner and illuminate important areas of unmet need. Furthermore, our findings make important and unique contributions to existing literature about the key “drivers” of service use across multiple sectors and underscore the importance of poor health in explaining service use across all sectors.

Although the social expectation is for women to leave abusive partners, and the Canadian public has come to expect that support will be provided for women to do so, our findings show that despite high service use rates, most women had difficulty getting the support they needed, with the greatest unmet need noted in key *health services* (i.e., dentist, family doctor, psychiatrist, psychologist). Importantly, general barriers to service access were primarily *structural*, and reflected both the depth of women’s economic challenges and a lack of service availability. Despite the intention of the Canadian health care system to be equitable and accessible and a modest social safety net in Canada, inability to pay for services, transportation problems, and lack of childcare collectively accounted for a significant proportion of the barriers faced, with low income women facing particular disadvantages in terms of access. These findings underscore the underlying financial strain associated with both violence and leaving an abusive partner within a social context characterized by a persistent gender-wage and income gap (Conference Board of Canada 2012; Statistics Canada 2011b). The remaining access barriers (i.e., no service available, lack of response from service providers, difficulty getting information, or being placed on a waiting list instead of receiving service) suggest inadequate services. These findings are particularly striking given that health and social care in Canada has been constructed as a social right (National Forum on Health 1997). However, the health care system has been designed to promote universal access to core *medical services*, such that some health services which are critical for women who have experienced IPV (such as access to dentists and counseling), are either not covered, or there is only partial coverage for a restricted set of services for particular groups of women (e.g., those receiving social assistance). These are important, but often unrecognized, service gaps which are fundamentally at odds with both social values and existing policy.

Our findings that women who had poorer physical and mental health were more likely to use services in *all* sectors we examined, suggest that these women may have greater need for support and assistance. There is growing evidence that the negative health impacts of IPV may be longstanding (Adkins and Kamp Dush 2010; Campbell and Soeken 1999; Wuest et al. 2007), yet poorer mental and physical health seldom have been identified as indicators of relative disadvantage and consequent need for support among women attempting to separate from abusive partners (Ford-Gilboe et al. 2005; Wuest et al. 2003). The high levels of health service use observed among women in poorer health may also reflect the challenges they face in accessing *appropriate* care. Our previous analysis of WHES data suggests that the health services women use may not meet their needs. For example, although 48 % of women experienced symptoms consistent with PTSD, only 7 % of women in this sample had received this diagnosis; rates of chronic disabling pain were twice that of Canadian women, but the use of prescription pain medication was not higher (Wuest et al. 2008).

Furthermore, disability related to chronic pain is more likely in women with a history of IPV than those without (Coker et al. 2005). In general populations, such disability interferes with physical functioning, social participation, and ability to work; prescription medication has been found ineffective in symptom management (Breivik et al. 2006). Although high levels of chronic pain, depression, and PTSD were unique predictors of using particular types of services in this analysis, these findings should be understood in light of the co-morbidity of these health problems. The extent to which health problems are *disabling* and interfere with a woman’s quality of life may better explain higher rates of service use, than the characteristics of particular health problems per se. In previous analyses of WHES data, symptoms of PTSD and depression were found to: a) be associated with high disability chronic pain (Wuest et al. 2008) and, b) mediate the impact of severity of lifetime abuse on chronic pain severity (Wuest et al. 2010). The current finding that high disability chronic pain is a predictor of general health, mental health, and social service use may also reflect management of chronic pain in isolation from mental health issues, economic situations, and abuse history. Treatment of depression and/or PTSD symptoms and validation of abuse history in the management of chronic pain may have an impact on patterns of health service use. Likewise, supporting women to improve their economic situations may reduce symptoms by reducing other sources of stress and increase options for self-management. It is not surprising that depressive symptoms predict greater use of mental health services. An area for future research is the finding that symptoms of PTSD predict use of legal services. Exploration of associations between patterns of trauma and engagement in criminal and family court systems is necessary to understand this result.

Our findings also suggest that women’s economic status affects their patterns of service use in complex ways. Fifty-nine percent of women in this sample had incomes that were below the poverty line and the mean family income was modest, suggesting an *overall* pattern of *economic disadvantage* in comparison to women in the general population. Our findings are consistent with previous research which shows that women who lack financial resources face more service barriers and have fewer viable options (Macy et al. 2005). The high use of some types of services in our study may reflect the services that women with few financial resources are *able* to access. For example, our finding that emergency service use was 20 times higher in our sample than in the general population is consistent with research showing higher income is associated with rapid access to health care, access to specialists, and to primary health care alternatives to Emergency use, and to shorter wait time for these services (Schoen et al. 2010). The fact that the poorest women in our sample were more likely to access both social and violence-specific services may reflect the fact that they have the greatest need for basic resources (e.g., food, safety, housing, support, income support) which these services provide at no cost. In particular, that 40 % of women were still accessing violence-specific services an average of 20 months after separation suggests that support from domestic violence services is required well beyond the crisis of leaving. Additionally, shelters and domestic violence services are grounded in philosophies of inclusion and often define their core services to include support for system navigation (or “advocacy”) and counselling (Wathen et al. 2015). There is strong evidence that domestic violence advocacy (i.e., information and support to deal with abuse and to access needed services) results in improvements in quality of life, safety actions, social support, access to services, and a reduction in violence (Ramsay et al. 2009), yet research is needed to more fully understand the context and outcomes of violence-specific services.

Our results suggest that mothers of dependent children were more likely to use social and legal services than women who did not have minor children living with them or who were not parents. In the context of IPV, previous research has shown that mothers’ actions are often motivated by a desire to protect their children and that they often fear losing custody if ex-partners succeed in portraying them as incapable of caring for their children (Varcoe and Irwin 2004; Wuest et al. 2004). Seeking help from “the system” is a calculated risk undertaken by some to gain needed support and control and prove their worth, while limiting ongoing interference in their lives (Ford-Gilboe et al. 2005). Even within publicly funded systems, some services also may be more accessible to mothers, either because service mandates are narrow (e.g., child protection), because eligibility criteria favor “families” over single adults (e.g., social assistance, public housing), or because service providers are influenced by societal norms which place higher social value on women who are mothers. Women who have experienced violence are a heterogeneous group; while barriers to access have consistently been identified, more research is needed to examine the ways in which multiple and intersecting layers of disadvantage play out in women’s ability to access, and benefit from, a wide range of services.

## Conclusions and Implications

The results of this cross-sectional analysis suggest associations between aspects of women’s abuse histories, social location, and health, and their use of services; longitudinal analyses are needed to identify whether service use changes over time after separation and to identify the mechanisms which explain such change. While our study points to varied predictors of service use across sectors, we did not find that severity of IPV independently predicted service use. However, this may be because IPV exerts indirect, rather than direct, effects on service use through its influence on other variables, such as health or social location. Studies which examine the ways in which women’s economic, social, and personal resources interact to influence service use will further enhance understanding of the complex ways in which the conditions of women’s lives shape their use of services. There is a need to investigate patterns of service use among: a) women experiencing abuse and living with an abusive partner; b) women experiencing abuse after separation; and, c) women who are separated and no longer experiencing abuse. Although understanding patterns of service use is helpful in identifying gaps in the system, the question of service quality and its impact on health and quality of life must also be considered. Future research should address whether services obtained by women actually meet their needs.

A number of implications for policy and practice can be drawn from the findings of this study. First, it is important to recognize that while publicly funded core services promote universal access, they also have gaps and weaknesses. Investment of adequate resources is needed to greatly reduce or eliminate modifiable access barriers such as lack of transportation or childcare, the costs of services or waiting lists, particularly for low income women who face the greatest barriers. For example, women should routinely be offered assistance with transportation and childcare and, where feasible, given priority access to services. Both dental care and counselling should be understood as core health services worthy of public funding. Furthermore, the need for income protection, guaranteed minimum incomes, and livable social assistance rates have been argued for the general Canadian population (Townson 2005). Such reforms are likely to have an especially important impact on women who have experienced violence, helping them improve their access to resources, and, ultimately, their longer term economic independence and health.

While services for abused women tend to focus on the crisis of leaving, our findings suggest that system capacity should reflect the long-term needs of women exposed to IPV, regardless of whether the abuse is ongoing or not. Since poor health predicted use of services in every sector, efforts to strengthen the quality of health services available to women who have experienced IPV are warranted. There is an urgent need to critically examine women’s access to primary health care services and to develop and test the impact of alternative models of service delivery on barriers and timely access.

Given women’s use of multiple services, an integrated, collaborative model of service delivery may be a more seamless and cost-effective way of delivering these services to women over time. Whether services and policies which focus directly on improving women’s health and economic situations lead to less reliance on formal support over time, and reduce the personal and system costs of IPV, merits further study. Embracing a *trauma and violence-informed* approach in service delivery, which is based on an understanding of the effects of abuse on people’s lives, health, and development (Elliot et al. 2005; Hopper et al. 2010) could improve the quality of services provided to women (Covington et al. 2008). Such an approach focuses on enhancing *emotional safety* in all sectors and begins from the position that it is not necessary to know whether those who seek services have experienced violence and/or trauma in their lives. Hence, a trauma- and violence-informed approach shifts system accountability for supporting women who have experienced violence beyond basic social services or mental health services, and toward all services and sectors where women may seek support for their healing.
